# Tetra-*n*-butyl­ammonium bis­(2,2-dicyano­ethyl­ene-1,1-dithiol­ato)palladium(II)

**DOI:** 10.1107/S1600536808037616

**Published:** 2008-11-20

**Authors:** Nyasha Kanganga, Kent R. Mann, Daron E. Janzen

**Affiliations:** a College of St Catherine, St Paul, Minnesota 55105, USA; b University of Minnesota, Minneapolis, Minnesota 55455, USA

## Abstract

In the title compound, (C_16_H_36_N)_2_[Pd(C_4_N_2_S_2_)_2_], the Pd^II^ center adopts a distorted square-planar geometry due to the four-membered chelate rings formed by coordination of the 2,2-dicyano­ethyl­ene-1,1-dithiol­ate (*i*-mnt) ligands [bite angle 75.0159 (17)°]. The bond distances in the coordinated *i*-mnt ligands indicate some delocalization of the π-system.

## Related literature

For general background, see: Fackler & Coucouvanis (1966 ▶); Werden *et al.* (1966 ▶). For related structures, see: Cao *et al.* (1999 ▶); Dong *et al.* (2005 ▶); Gao *et al.* (2005 ▶); Long *et al.* (1996 ▶, 1997 ▶, 1998 ▶); Mori *et al.* (1995 ▶); Sun *et al.* (2006 ▶); Zhu *et al.* (1991 ▶).
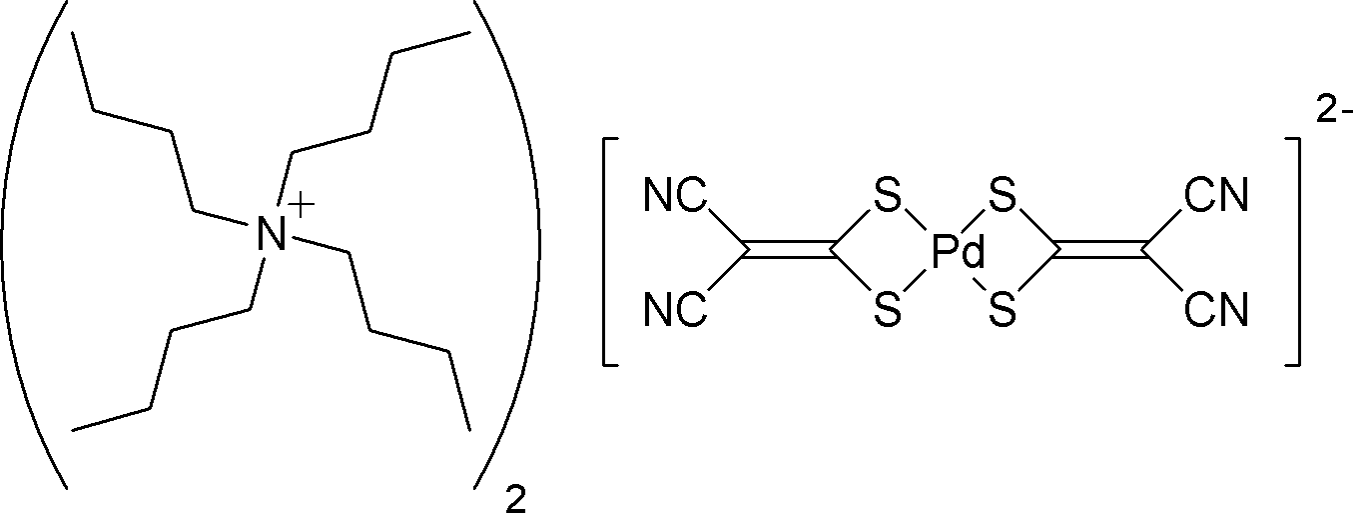


## Experimental

### 

#### Crystal data

(C_16_H_36_N)_2_[Pd(C_4_N_2_S_2_)_2_]
*M* *_r_* = 871.68
Monoclinic, 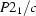

*a* = 13.9468 (13) Å
*b* = 8.6267 (8) Å
*c* = 20.3231 (19) Å
β = 108.218 (2)°
*V* = 2322.6 (4) Å^3^
*Z* = 2
Mo *K*α radiation
μ = 0.61 mm^−1^
*T* = 173 (2) K
0.50 × 0.40 × 0.33 mm

#### Data collection

Bruker SMART CCD area-detector diffractometer
Absorption correction: multi-scan (*SADABS*; Bruker 2003 ▶) *T*_min_ = 0.749, *T*_max_ = 0.824
22206 measured reflections
4124 independent reflections
3613 reflections with *I* > 2σ(*I*)
*R*_int_ = 0.027

#### Refinement

*R*[*F* ^2^ > 2σ(*F* ^2^)] = 0.024
*wR*(*F* ^2^) = 0.063
*S* = 1.02
4124 reflections
236 parameters
H-atom parameters constrained
Δρ_max_ = 0.24 e Å^−3^
Δρ_min_ = −0.28 e Å^−3^

### 

Data collection: *SMART* (Bruker, 2003 ▶); cell refinement: *SAINT* (Bruker, 2006 ▶); data reduction: *SAINT*; program(s) used to solve structure: *SHELXTL* (Sheldrick, 2008 ▶); program(s) used to refine structure: *SHELXTL*; molecular graphics: *SHELXTL*; software used to prepare material for publication: *SHELXTL*.

## Supplementary Material

Crystal structure: contains datablocks I, global. DOI: 10.1107/S1600536808037616/pk2131sup1.cif

Structure factors: contains datablocks I. DOI: 10.1107/S1600536808037616/pk2131Isup2.hkl

Additional supplementary materials:  crystallographic information; 3D view; checkCIF report

## Figures and Tables

**Fig. 1.**
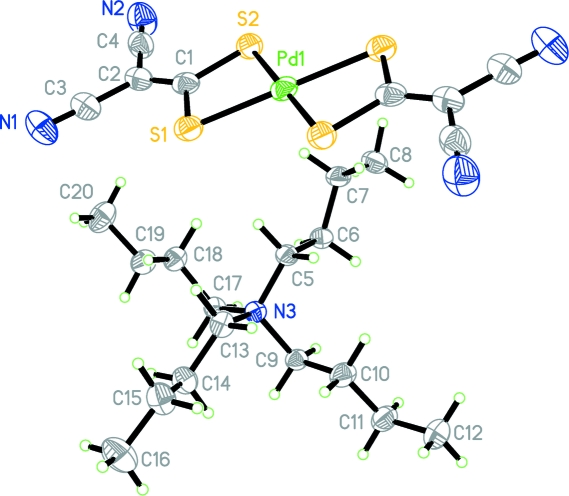
The molecular structure of (C16H36N)2[Pd(S2C4N2)2] showing 50% displacement ellipsoids for the non-hydrogen atoms. Only the crystallographically independent atoms are labelled.

## supplementary crystallographic information

### Comment

Salts of metal complexes of [Pd(*i*-mnt)_2_]^2-^ (*i*-mnt = 2,2-dicyanoethylene-1,1-dithiolate) have been studied for their interesting electronic properties including their use in conducting charge-transfer salts (Mori *et al.*, 1995; Zhu *et al.*, 1991) and their redox behavior especially in relation to the analagous isomeric ligand 1,2-dicyanoethylene-1,2-dithiolate (mnt^2-^) complexes (Fackler & Coucouvanis, 1966; Werden *et al.*, 1966). In sharp contrast to mnt complexes of the form [*M*(mnt)_2_]^2-^ (*M*= Ni^II^, Pd^II^, Pt^II^) which do exhibit reversible oxidation behavior, analagous *i*-mnt complexes of the form [*M*(*i*-mnt)_2_]^2-^ do not. This effect is attributed to better π-delocalization of the five-membered rings formed by complexation of mnt compared with four-membered chelate rings of *i*-mnt complexes. Salts of [Pd(*i*-mnt)_2_]^2-^ have also been studied as supramolecular linker groups in organic-inorganic hybrid coordination polymers (Cao *et al.*, 1999; Dong *et al.*, 2005; Gao *et al.*, 2005; Long *et al*., 1997,1998; Sun *et al.*, 2006). While several *x*-ray structures of [Pd(*i*-mnt)_2_]^2-^ with alkali metal-complexed crown ether salts and one simple potassium salt have been reported, only one other simple non-coordinating cation salt (tetraethylammonium, Long *et al.* 1996) has been structurally characterized.

The structure of the anion in (C_16_H_36_N)_2_[Pd(S_2_C_4_N_2_)_2_] shows significant distortions from a square planar environment as forced by the four-membered chelate rings of the *i*-mnt ligands, with the *i*-mnt bite angle of S(2)—Pd(1)—S(1) 75.0159 (17)°. As the Pd sits on the inversion centre at (0,0,0) in the space group *P*2_1_/*c*, *Z*'=0.5, the anion is quite planar, with a calculated r.m.s. deviation from a least-squares plane formed by all atoms of the complex anion of 0.0806 (13) Å. The bond lengths within the coordinated *i*-mnt ligand, in particular the bonds C(1)—C(2) 1.379 (3) Å, C(2)—C(3) 1.424 (3) Å, and C(2)—C(4) 1.429 (3)Å are very similar to those observed in the tetraethylammonium salt, showing significant π-delocalization. No columnar stacking is observed amongst the complex anions. As expected, upon comparison of this structure with that of the tetraethylammonium salt, little effect was observed on the intramolecular features of the complex anion.

### Experimental

The title compound (C_16_H_36_N)_2_[Pd(S_2_C_4_N_2_)_2_] was prepared using a procedure similar to that described by Fackler and Coucouvanis (1966) substituting the use of tetra-n-propylammonium iodide with tetra-n-butylammonium bromide. The title compound has been previously characterized by Werden *et al.* (1966). Spectroscopic analysis of the title compound obtained by this procedure was consistent with the data previously reported. Crystals were obtained by diffusion of diethyl ether into a concentrated solution of the title compound dissolved in acetone.

### Refinement

The H atoms were geometrically placed (C—H = 0.98–0.99 Å) and refined as riding with *U*_iso_(H) = 1.2*U*_eq_(C) or 1.5*U*_eq_(methyl C).

### Crystal data

**Table:**

(C_16_H_36_N)_2_[Pd(C_4_N_2_S_2_)_2_]	*F*_000_ = 928
*M**_r_* = 871.68	*D*_x_ = 1.246 Mg m^−^^3^
Monoclinic, *P*2_1_/*c*	Mo *K*α radiation λ = 0.71073 Å
Hall symbol: -P 2ybc	Cell parameters from 3632 reflections
*a* = 13.9468 (13) Å	θ = 2.2–25.0º
*b* = 8.6267 (8) Å	µ = 0.61 mm^−^^1^
*c* = 20.3231 (19) Å	*T* = 173 (2) K
β = 108.218 (2)º	Block, orange
*V* = 2322.6 (4) Å^3^	0.50 × 0.40 × 0.33 mm
*Z* = 2	

### Data collection

**Table:**

Bruker SMART CCD area-detector diffractometer	4124 independent reflections
Radiation source: normal-focus sealed tube	3613 reflections with *I* > 2σ(*I*)
Monochromator: graphite	*R*_int_ = 0.027
*T* = 173(2) K	θ_max_ = 25.1º
φ scans	θ_min_ = 1.5º
Absorption correction: multi-scan(SADABS; Bruker 2003)	*h* = −16→16
*T*_min_ = 0.749, *T*_max_ = 0.824	*k* = −10→10
22206 measured reflections	*l* = −24→24

### Refinement

**Table:**

Refinement on *F*^2^	Secondary atom site location: difference Fourier map
Least-squares matrix: full	Hydrogen site location: inferred from neighbouring sites
*R*[*F*^2^ > 2σ(*F*^2^)] = 0.024	H-atom parameters constrained
*wR*(*F*^2^) = 0.063	*w* = 1/[σ^2^(*F*_o_^2^) + (0.0322*P*)^2^ + 0.8279*P*] where *P* = (*F*_o_^2^ + 2*F*_c_^2^)/3
*S* = 1.02	(Δ/σ)_max_ = 0.001
4124 reflections	Δρ_max_ = 0.25 e Å^−^^3^
236 parameters	Δρ_min_ = −0.28 e Å^−^^3^
Primary atom site location: structure-invariant direct methods	Extinction correction: none

### Special details

**Table:**

Geometry. All e.s.d.'s (except the e.s.d. in the dihedral angle between two l.s. planes) are estimated using the full covariance matrix. The cell e.s.d.'s are taken into account individually in the estimation of e.s.d.'s in distances, angles and torsion angles; correlations between e.s.d.'s in cell parameters are only used when they are defined by crystal symmetry. An approximate (isotropic) treatment of cell e.s.d.'s is used for estimating e.s.d.'s involving l.s. planes.
Refinement. Refinement of *F*^2^ against ALL reflections. The weighted *R*-factor *wR* and goodness of fit *S* are based on *F*^2^, conventional *R*-factors *R* are based on *F*, with *F* set to zero for negative *F*^2^. The threshold expression of *F*^2^ > σ(*F*^2^) is used only for calculating *R*-factors(gt) *etc*. and is not relevant to the choice of reflections for refinement. *R*-factors based on *F*^2^ are statistically about twice as large as those based on *F*, and *R*- factors based on ALL data will be even larger.

### Fractional atomic coordinates and isotropic or equivalent isotropic displacement parameters (Å^2^)

**Table:**

	*x*	*y*	*z*	*U*_iso_*/*U*_eq_	
Pd1	0.0000	1.0000	1.0000	0.03612 (8)	
S1	0.15463 (4)	1.09186 (5)	0.99538 (2)	0.04065 (12)	
C1	0.19356 (14)	1.09487 (19)	1.08491 (9)	0.0367 (4)	
N1	0.40960 (13)	1.2828 (2)	1.07224 (9)	0.0542 (5)	
S2	0.10006 (4)	1.02365 (5)	1.11567 (3)	0.04049 (12)	
N2	0.33826 (14)	1.1436 (2)	1.25846 (10)	0.0597 (5)	
C2	0.28567 (14)	1.1511 (2)	1.12541 (9)	0.0375 (4)	
N3	0.25990 (9)	0.61333 (15)	0.92368 (7)	0.0254 (3)	
C3	0.35433 (15)	1.2222 (2)	1.09551 (10)	0.0415 (5)	
C4	0.31446 (15)	1.1474 (2)	1.19936 (11)	0.0438 (5)	
C5	0.17965 (12)	0.64663 (19)	0.95836 (8)	0.0280 (4)	
H5A	0.1898	0.7533	0.9772	0.034*	
H5B	0.1125	0.6430	0.9226	0.034*	
C6	0.17926 (13)	0.53612 (19)	1.01639 (9)	0.0312 (4)	
H6A	0.2494	0.5187	1.0465	0.037*	
H6B	0.1510	0.4350	0.9965	0.037*	
C7	0.11676 (13)	0.6011 (2)	1.05940 (9)	0.0338 (4)	
H7A	0.1484	0.6980	1.0823	0.041*	
H7B	0.0484	0.6270	1.0285	0.041*	
C8	0.10805 (15)	0.4871 (2)	1.11407 (10)	0.0402 (4)	
H8A	0.0707	0.5352	1.1423	0.060*	
H8B	0.1757	0.4583	1.1437	0.060*	
H8C	0.0721	0.3942	1.0915	0.060*	
C9	0.25005 (12)	0.44804 (19)	0.89542 (8)	0.0278 (4)	
H9A	0.3061	0.4288	0.8763	0.033*	
H9B	0.2585	0.3754	0.9345	0.033*	
C10	0.15178 (13)	0.4104 (2)	0.84001 (10)	0.0379 (4)	
H10A	0.1458	0.4731	0.7981	0.045*	
H10B	0.0944	0.4367	0.8568	0.045*	
C11	0.14795 (15)	0.2387 (2)	0.82185 (10)	0.0413 (4)	
H11A	0.2031	0.2147	0.8025	0.050*	
H11B	0.1592	0.1769	0.8646	0.050*	
C12	0.04792 (16)	0.1920 (3)	0.76986 (12)	0.0587 (6)	
H12A	0.0489	0.0809	0.7600	0.088*	
H12B	0.0372	0.2510	0.7269	0.088*	
H12C	−0.0069	0.2140	0.7891	0.088*	
C13	0.24560 (12)	0.7323 (2)	0.86637 (8)	0.0302 (4)	
H13A	0.1768	0.7201	0.8333	0.036*	
H13B	0.2496	0.8370	0.8870	0.036*	
C14	0.32053 (13)	0.7239 (2)	0.82619 (9)	0.0364 (4)	
H14A	0.3276	0.6150	0.8131	0.044*	
H14B	0.3874	0.7602	0.8559	0.044*	
C15	0.28591 (14)	0.8236 (2)	0.76111 (9)	0.0434 (5)	
H15A	0.2205	0.7840	0.7306	0.052*	
H15B	0.2754	0.9312	0.7743	0.052*	
C16	0.36177 (18)	0.8238 (3)	0.72159 (12)	0.0618 (6)	
H16A	0.3374	0.8910	0.6809	0.093*	
H16B	0.3702	0.7180	0.7067	0.093*	
H16C	0.4268	0.8625	0.7516	0.093*	
C17	0.36558 (11)	0.62498 (19)	0.97581 (8)	0.0271 (3)	
H17A	0.3757	0.5354	1.0077	0.033*	
H17B	0.4152	0.6155	0.9503	0.033*	
C18	0.38904 (12)	0.7716 (2)	1.01894 (9)	0.0323 (4)	
H18A	0.3397	0.7849	1.0445	0.039*	
H18B	0.3843	0.8627	0.9885	0.039*	
C19	0.49551 (13)	0.7591 (2)	1.06987 (9)	0.0375 (4)	
H19A	0.4987	0.6693	1.1008	0.045*	
H19B	0.5435	0.7399	1.0438	0.045*	
C20	0.52758 (15)	0.9040 (2)	1.11359 (11)	0.0516 (5)	
H20A	0.5969	0.8913	1.1444	0.077*	
H20B	0.4823	0.9207	1.1413	0.077*	
H20C	0.5242	0.9935	1.0833	0.077*	

### Atomic displacement parameters (Å^2^)

**Table:**

	*U*^11^	*U*^22^	*U*^33^	*U*^12^	*U*^13^	*U*^23^
Pd1	0.05110 (14)	0.02259 (11)	0.03883 (13)	0.00135 (8)	0.02006 (10)	−0.00010 (8)
S1	0.0562 (3)	0.0330 (2)	0.0383 (3)	0.0008 (2)	0.0228 (2)	0.00116 (19)
C1	0.0513 (11)	0.0244 (9)	0.0408 (10)	0.0087 (8)	0.0236 (9)	0.0046 (7)
N1	0.0447 (10)	0.0646 (12)	0.0576 (11)	0.0134 (9)	0.0224 (9)	0.0267 (9)
S2	0.0536 (3)	0.0341 (3)	0.0400 (3)	−0.0036 (2)	0.0237 (2)	0.00052 (19)
N2	0.0626 (12)	0.0758 (14)	0.0422 (11)	−0.0116 (10)	0.0185 (9)	0.0066 (9)
C2	0.0451 (11)	0.0344 (10)	0.0376 (10)	0.0092 (8)	0.0197 (9)	0.0077 (8)
N3	0.0217 (7)	0.0270 (7)	0.0260 (7)	0.0027 (5)	0.0054 (6)	−0.0010 (6)
C3	0.0414 (11)	0.0426 (11)	0.0419 (11)	0.0145 (9)	0.0149 (9)	0.0148 (9)
C4	0.0453 (11)	0.0418 (11)	0.0486 (13)	0.0000 (9)	0.0209 (10)	0.0068 (9)
C5	0.0226 (8)	0.0282 (8)	0.0331 (9)	0.0038 (7)	0.0085 (7)	−0.0034 (7)
C6	0.0307 (9)	0.0273 (9)	0.0378 (10)	0.0033 (7)	0.0138 (8)	−0.0023 (7)
C7	0.0299 (9)	0.0350 (10)	0.0376 (10)	0.0043 (7)	0.0120 (8)	−0.0073 (8)
C8	0.0382 (10)	0.0494 (12)	0.0377 (10)	0.0005 (9)	0.0188 (8)	−0.0042 (8)
C9	0.0269 (8)	0.0267 (8)	0.0294 (9)	0.0027 (7)	0.0083 (7)	−0.0022 (7)
C10	0.0293 (9)	0.0410 (11)	0.0402 (10)	0.0017 (8)	0.0062 (8)	−0.0107 (8)
C11	0.0508 (12)	0.0384 (10)	0.0344 (10)	−0.0087 (9)	0.0126 (9)	−0.0071 (8)
C12	0.0543 (13)	0.0669 (15)	0.0559 (13)	−0.0249 (12)	0.0187 (11)	−0.0259 (12)
C13	0.0278 (9)	0.0300 (9)	0.0289 (9)	0.0024 (7)	0.0034 (7)	0.0033 (7)
C14	0.0345 (10)	0.0385 (10)	0.0363 (10)	0.0008 (8)	0.0112 (8)	0.0038 (8)
C15	0.0409 (11)	0.0538 (12)	0.0330 (10)	−0.0038 (9)	0.0078 (8)	0.0069 (9)
C16	0.0645 (15)	0.0790 (17)	0.0487 (13)	0.0003 (13)	0.0276 (12)	0.0143 (12)
C17	0.0211 (8)	0.0311 (9)	0.0269 (8)	0.0029 (7)	0.0042 (7)	0.0007 (7)
C18	0.0269 (9)	0.0317 (9)	0.0343 (10)	0.0012 (7)	0.0036 (7)	−0.0024 (7)
C19	0.0297 (9)	0.0397 (10)	0.0376 (10)	0.0024 (8)	0.0024 (8)	−0.0049 (8)
C20	0.0426 (11)	0.0459 (12)	0.0541 (13)	−0.0044 (9)	−0.0025 (10)	−0.0090 (10)

### Geometric parameters (Å, °)

**Table:**

Pd1—S1^i^	2.3269 (5)	C10—H10B	0.9900
Pd1—S1	2.3269 (5)	C11—C12	1.518 (3)
Pd1—S2^i^	2.3375 (5)	C11—H11A	0.9900
Pd1—S2	2.3375 (5)	C11—H11B	0.9900
S1—C1	1.7288 (19)	C12—H12A	0.9800
C1—C2	1.379 (3)	C12—H12B	0.9800
C1—S2	1.7258 (19)	C12—H12C	0.9800
N1—C3	1.148 (2)	C13—C14	1.516 (2)
N2—C4	1.142 (2)	C13—H13A	0.9900
C2—C3	1.424 (3)	C13—H13B	0.9900
C2—C4	1.429 (3)	C14—C15	1.524 (2)
N3—C13	1.518 (2)	C14—H14A	0.9900
N3—C5	1.525 (2)	C14—H14B	0.9900
N3—C17	1.5260 (19)	C15—C16	1.515 (3)
N3—C9	1.527 (2)	C15—H15A	0.9900
C5—C6	1.518 (2)	C15—H15B	0.9900
C5—H5A	0.9900	C16—H16A	0.9800
C5—H5B	0.9900	C16—H16B	0.9800
C6—C7	1.521 (2)	C16—H16C	0.9800
C6—H6A	0.9900	C17—C18	1.515 (2)
C6—H6B	0.9900	C17—H17A	0.9900
C7—C8	1.517 (3)	C17—H17B	0.9900
C7—H7A	0.9900	C18—C19	1.525 (2)
C7—H7B	0.9900	C18—H18A	0.9900
C8—H8A	0.9800	C18—H18B	0.9900
C8—H8B	0.9800	C19—C20	1.517 (3)
C8—H8C	0.9800	C19—H19A	0.9900
C9—C10	1.512 (2)	C19—H19B	0.9900
C9—H9A	0.9900	C20—H20A	0.9800
C9—H9B	0.9900	C20—H20B	0.9800
C10—C11	1.523 (3)	C20—H20C	0.9800
C10—H10A	0.9900		
			
S1^i^—Pd1—S1	180.0	C12—C11—H11A	109.1
S1^i^—Pd1—S2^i^	75.015 (17)	C10—C11—H11A	109.1
S1—Pd1—S2^i^	104.985 (17)	C12—C11—H11B	109.1
S1^i^—Pd1—S2	104.985 (17)	C10—C11—H11B	109.1
S1—Pd1—S2	75.015 (17)	H11A—C11—H11B	107.8
S2^i^—Pd1—S2	180.0	C11—C12—H12A	109.5
C1—S1—Pd1	87.29 (7)	C11—C12—H12B	109.5
C2—C1—S2	125.26 (14)	H12A—C12—H12B	109.5
C2—C1—S1	124.12 (14)	C11—C12—H12C	109.5
S2—C1—S1	110.59 (11)	H12A—C12—H12C	109.5
C1—S2—Pd1	87.02 (7)	H12B—C12—H12C	109.5
C1—C2—C3	121.47 (17)	C14—C13—N3	115.69 (13)
C1—C2—C4	121.52 (17)	C14—C13—H13A	108.4
C3—C2—C4	116.93 (18)	N3—C13—H13A	108.4
C13—N3—C5	106.73 (12)	C14—C13—H13B	108.4
C13—N3—C17	110.78 (12)	N3—C13—H13B	108.4
C5—N3—C17	110.85 (12)	H13A—C13—H13B	107.4
C13—N3—C9	111.59 (12)	C13—C14—C15	110.84 (15)
C5—N3—C9	110.92 (12)	C13—C14—H14A	109.5
C17—N3—C9	106.04 (11)	C15—C14—H14A	109.5
N1—C3—C2	178.4 (2)	C13—C14—H14B	109.5
N2—C4—C2	179.3 (2)	C15—C14—H14B	109.5
C6—C5—N3	114.98 (13)	H14A—C14—H14B	108.1
C6—C5—H5A	108.5	C16—C15—C14	112.12 (17)
N3—C5—H5A	108.5	C16—C15—H15A	109.2
C6—C5—H5B	108.5	C14—C15—H15A	109.2
N3—C5—H5B	108.5	C16—C15—H15B	109.2
H5A—C5—H5B	107.5	C14—C15—H15B	109.2
C5—C6—C7	110.90 (14)	H15A—C15—H15B	107.9
C5—C6—H6A	109.5	C15—C16—H16A	109.5
C7—C6—H6A	109.5	C15—C16—H16B	109.5
C5—C6—H6B	109.5	H16A—C16—H16B	109.5
C7—C6—H6B	109.5	C15—C16—H16C	109.5
H6A—C6—H6B	108.0	H16A—C16—H16C	109.5
C8—C7—C6	111.91 (14)	H16B—C16—H16C	109.5
C8—C7—H7A	109.2	C18—C17—N3	116.33 (13)
C6—C7—H7A	109.2	C18—C17—H17A	108.2
C8—C7—H7B	109.2	N3—C17—H17A	108.2
C6—C7—H7B	109.2	C18—C17—H17B	108.2
H7A—C7—H7B	107.9	N3—C17—H17B	108.2
C7—C8—H8A	109.5	H17A—C17—H17B	107.4
C7—C8—H8B	109.5	C17—C18—C19	108.79 (14)
H8A—C8—H8B	109.5	C17—C18—H18A	109.9
C7—C8—H8C	109.5	C19—C18—H18A	109.9
H8A—C8—H8C	109.5	C17—C18—H18B	109.9
H8B—C8—H8C	109.5	C19—C18—H18B	109.9
C10—C9—N3	115.76 (13)	H18A—C18—H18B	108.3
C10—C9—H9A	108.3	C20—C19—C18	112.68 (15)
N3—C9—H9A	108.3	C20—C19—H19A	109.1
C10—C9—H9B	108.3	C18—C19—H19A	109.1
N3—C9—H9B	108.3	C20—C19—H19B	109.1
H9A—C9—H9B	107.4	C18—C19—H19B	109.1
C9—C10—C11	110.14 (15)	H19A—C19—H19B	107.8
C9—C10—H10A	109.6	C19—C20—H20A	109.5
C11—C10—H10A	109.6	C19—C20—H20B	109.5
C9—C10—H10B	109.6	H20A—C20—H20B	109.5
C11—C10—H10B	109.6	C19—C20—H20C	109.5
H10A—C10—H10B	108.1	H20A—C20—H20C	109.5
C12—C11—C10	112.46 (17)	H20B—C20—H20C	109.5
			
S2^i^—Pd1—S1—C1	178.05 (6)	C5—C6—C7—C8	175.31 (15)
S2—Pd1—S1—C1	−1.95 (6)	C13—N3—C9—C10	−56.92 (18)
Pd1—S1—C1—C2	−175.49 (15)	C5—N3—C9—C10	61.94 (18)
Pd1—S1—C1—S2	2.72 (8)	C17—N3—C9—C10	−177.64 (15)
C2—C1—S2—Pd1	175.47 (16)	N3—C9—C10—C11	−174.36 (14)
S1—C1—S2—Pd1	−2.71 (8)	C9—C10—C11—C12	176.18 (16)
S1^i^—Pd1—S2—C1	−178.05 (6)	C5—N3—C13—C14	178.03 (14)
S1—Pd1—S2—C1	1.95 (6)	C17—N3—C13—C14	57.26 (18)
S2—C1—C2—C3	−173.49 (14)	C9—N3—C13—C14	−60.64 (18)
S1—C1—C2—C3	4.5 (3)	N3—C13—C14—C15	167.77 (15)
S2—C1—C2—C4	3.4 (3)	C13—C14—C15—C16	177.23 (17)
S1—C1—C2—C4	−178.64 (14)	C13—N3—C17—C18	67.16 (18)
C13—N3—C5—C6	178.31 (14)	C5—N3—C17—C18	−51.13 (18)
C17—N3—C5—C6	−60.97 (17)	C9—N3—C17—C18	−171.60 (14)
C9—N3—C5—C6	56.56 (17)	N3—C17—C18—C19	177.49 (14)
N3—C5—C6—C7	165.40 (13)	C17—C18—C19—C20	177.67 (17)

Symmetry codes: (i) −*x*, −*y*+2, −*z*+2.
